# Editorial: Multiple Sclerosis – From Bench to Bedside: Currents Insights Into Pathophysiological Concepts and Their Potential Impact on Patients

**DOI:** 10.3389/fimmu.2020.00137

**Published:** 2020-02-07

**Authors:** Paulus S. Rommer, Martin S. Weber, Zsolt Illes, Uwe K. Zettl

**Affiliations:** ^1^Department of Neurology, Medical University of Vienna, Vienna, Austria; ^2^Medical Institute of Neuropathology, University Medical Center, Göttingen, Germany; ^3^Department of Neurology, University Medical Center, Göttingen, Germany; ^4^Department of Neurology, Odense University Hospital, Odense, Denmark; ^5^Institute of Clinical Research, University of Southern Denmark, Odense, Denmark; ^6^Department of Neurology, Neuroimmunological Section, University of Rostock, Rostock, Germany

**Keywords:** multiple sclerosis, multiple sclerosis and neuroimmunology, drug therapy, epidemiology, etiology

More than 2 million patients worldwide suffer from multiple sclerosis (MS) (1), and it is the most common cause of disability in young adulthood. Different disease courses are described, possibly reflecting different pathophysiological scenarios. Although treatment options for MS have changed dramatically in recent decades, the cause of the disease is still unknown (2, 3).

The special topic “Multiple Sclerosis—From Bench to Bedside: Currents Insights into Pathophysiological Concepts and Their Potential Impact on Patients” deals with diverse aspects of MS, and contains 22 articles that approach MS from different angles.

Findings from histopathological studies have shown that different immune cells also play a role in different disease courses. The significance of B and T cells in these various disease courses were summarized in a review (Lassmann). The importance of B cells was also highlighted in neuromyelitis optica spectrum disorder (NMOSD) and related diseases (Häusser-Kinzel and Weber). In a study, Faigle et al. examined the role of citrullinated peptides identified in the MS brain tissue, and concluded that citrullination may not be important for the activation of T cells, but could be the consequence of the inflammatory cascade. Di Pauli and Berger reviewed antibody diagnostics and discussed the clinical presentation and pathology of MOG-antibody disease. Zhong et al. examined differential diagnostic questions of MOG-antibody disease in connection with epilepsy and encephalopathy.

Animal models can provide new insights into immunological processes, although findings should be extrapolated to MS with caution. In two experimental autoimmune encephalomyelitis (EAE) models of progressive MS—one with T-cell infiltration in the CNS and one without—cytokines and transcriptomes were identified as potential candidates for biomarkers by means of bioinformatic analyses (Omura et al.). The importance of synapses in the neuronal network and their function and possible interventions were investigated in an EAE study by LoPresti. Another animal study showed that carnosol inhibits Th17 cells and may be a potential candidate for the treatment of MS (Li et al.). Connexin was identified as a possible modulator of microglia activity in EAE (Fang et al.).

The analysis of the cerebrospinal fluid (CSF) is important for establishing diagnosis, distinguishing MS from other diseases, and obtaining information about immunological processes within the CNS environment. Deisenhammer et al. summarized the most important questions about the CSF in MS, its importance, but also limitations, and potential novel biomarkers. The distinction between MS and other autoimmune diseases, particularly rheumatological diseases, is often difficult. Venhoff et al. examined 108 patients suffering from rheumatological diseases with CNS involvement or MS, and investigated the significance of the measles virus, rubella virus, and varicella zoster virus reaction (MRZ)-reaction in differential diagnosis. It was found that in cases, where clear clinical separation was not possible, the MRZ reaction was useful in addition to oligoclonal bands (OCBs) and specific autoantibodies (Venhoff et al.).

Cytokines are part of the immunological cascade and their importance for inflammation and disease activity is undisputed. Redundant role and interconnection among a multitude of cytokines complicate interpretation. Computational intelligence could be one possibility for evaluation, and such approaches could help to use cytokine levels as prognostic markers (Goyal et al.; Omura et al.). Besides influencing disease activity, such as relapses and the progression of disability, cytokines can also play a role in the development of symptoms. Hanken et al. showed that fatigue and IL-1ß are linked and that disease-modifying treatments lead to a decrease in cytokine levels and an improvement in clinical symptoms.

The importance of environmental factors, the microbiome, aging, gender, and hormones appears to play a role in the susceptibility to MS. The relevance of epidemiological studies is undisputed and can help to demonstrate these relationships. These factors were considered in several articles (Ghareghani et al.; Krementsov et al.; Jiang et al.; Sena et al.).

The number of therapies for relapsing MS has increased in the recent decades, allowing a more personalized treatment approach after weighing, among others risks, efficacy, pregnancy issues, and convenience for patients. The mode of action and immunological effects of all approved treatments were highlighted in a review (Rommer et al.). In a longitudinal analysis, Hegen et al. showed that glatiramer acetate, interferon-beta, and natalizumab had no effect on the anti-JCV index. By introducing highly effective treatments, however, more attention has to be paid to the risk of infections and possible vaccinations. The extent to which vaccinations can protect against infections, or whether vaccination protection can be built up under the therapies, must be discussed in complex terms. There is scientific consensus that vaccinations cannot cause MS. Zrzavy et al. summarized known data on vaccinations. While the number of therapeutic options for the relapsing course has been significantly increased (Rommer et al.), the treatments for the progressive course are very limited. In a review by LoPresti, possible interventions for patients with a progressive course were discussed.

With a few exceptions, immunotherapies for MS are not approved during pregnancy. Registry data that investigate the effects of therapies on the unborn child are therefore of paramount importance. Using data from the Danish Multiple Sclerosis Registry, Andersen et al. studied the experience with teriflunomide in pregnant women.

The unparalleled growth in knowledge about MS has enabled a range of therapeutic options that was unthinkable just 20 years ago. The compelling data is causing a frenzied debate and the Internet is being flooded with questions about whether MS is curable. By definition, health is a state of complete physical, mental and social well-being and not merely the absence of disease or infirmity (www.who.int)^1^, and this definition has not changed since 1948. In this sense, we cannot achieve cure at present, but highly effective therapies provide options of no disease activity for years, and perhaps even ultimately in a subgroup of patients. First treatments for progressive disease has recently become available, and attention has been shifted to develop therapies for this type of MS. A possible future improvement that could be achieved in the near future would be a clearer stratification of treatment allocation to specific subgroups of patients. Such stratification would be of immediate benefit to all patients, as it would most likely drastically reduce the risk of treatment failure. An increasingly better understanding of the complex interactions in the human body, and interaction with the environment could be a basis for future developments. There is a need for better risk stratification, for further therapy options for progressive MS, for neuroprotection, and for improving quality of life—by reducing disease activity and providing increasingly diverse and effective symptomatic treatments. The cause of MS is and will probably remain unclear for some time to come, and the identification of such factors is a long-term goal of health, and therefore, to some extent, of cure.

## Author Contributions

PR, MW, ZI, and UZ involved in the process of guest editorship and contributed to the editorial and to the management of the whole special topic.

### Conflict of Interest

The authors declare that the research was conducted in the absence of any commercial or financial relationships that could be construed as a potential conflict of interest.

## Footnotes

^1^https://www.who.int/governance/eb/who_constitution_en.pdf (accessed December 8, 2019).
